# Marker-Assisted Gene Pyramiding and the Reliability of Using SNP Markers Located in the Recombination Suppressed Regions of Sunflower (*Helianthus annuus* L.)

**DOI:** 10.3390/genes11010010

**Published:** 2019-12-20

**Authors:** Lili Qi, Guojia Ma

**Affiliations:** 1 USDA-Agricultural Research Service, Edward T. Schafer Agricultural Research Center, 1616 Albrecht Blvd. N, Fargo, ND 58102-2765, USA; 2 Department of Plant Sciences, North Dakota State University, Fargo, ND 58108, USA; guojia.ma@ndsu.edu

**Keywords:** sunflower, rust, downy mildew, resistance, gene pyramiding

## Abstract

Rust caused by the fungus *Puccinia helianthi* and downy mildew (DM) caused by the obligate pathogen *Plasmopara halstedii* are two of the most globally important sunflower diseases. Resistance to rust and DM is controlled by race-specific single dominant genes. The present study aimed at pyramiding rust resistance genes combined with a DM resistance gene, using molecular markers. Four rust resistant lines, HA-R3 (carrying the *R_4_* gene), HA-R2 (*R_5_*), HA-R8 (*R_15_*), and RHA 397 (*R_13b_*), were each crossed with a common line, RHA 464, carrying a rust gene *R_12_* and a DM gene *Pl_Arg_*. An additional cross was made between HA-R8 and RHA 397. Co-dominant simple sequence repeat (SSR) and single nucleotide polymorphism (SNP) markers linked to the target genes were used to discriminate between homozygotes and heterozygotes in F_2_ populations. Five pyramids with different combinations of rust resistance genes were selected in the homozygous condition through marker-assisted selection, and three of them were combined with a DM resistance gene *Pl_Arg_*: *R_4_*/*R_12_*/*Pl_Arg_*, *R_5_*/*R_12_*/*Pl_Arg_*, *R_13b_*/*R_12_*/*Pl_Arg_*, *R_15_*/*R_12_*, and *R_13b_*/*R_15_*. The pyramiding lines with the stacking of two rust and one DM genes were resistant to all known races of North American sunflower rust and all known races of the pathogen causing DM, potentially providing multiple and durable resistance to both rust and DM. A cluster of 12 SNP markers spanning a region of 34.5 Mb on chromosome 1, which co-segregate with *Pl_Arg_*, were tested in four populations. Use of those markers, located in a recombination suppressed region in marker selection, is discussed.

## 1. Introduction

Sunflower (*Helianthus annuus* L.) is cultivated globally and is highly valued as a source of edible oil rich in lineoleic or oleic acids with a high vitamin E content. However, sunflower crops can be infected by disease-causing bacterial, fungal, and viral pathogens, subsequently reducing yield and quality. Rust caused by the fungus *Puccinia helianthi* Schwein. and downy mildew (DM) caused by the obligate pathogen *Plasmopara halstedii* (Farl.) Berl. et. de Toni are the two of the most important sunflower diseases. Both are native to North America (NA) but have spread to nearly every sunflower growing region in the world; for review see [1,2]. Resistance to rust and DM is controlled by race-specific single dominant genes. There is a long history of the use of resistant varieties and hybrids to control rust and DM in sunflower production.

The first rust resistant cultivar was developed in Canada in 1954, and subsequently, two rust resistance genes (*R* genes), *R_1_* and *R_2_*, were discovered [3,4]. Since then, a total of 13 rust *R* genes, *R_1_*–*R_5_*, *R_10_*–*R_15_*, *P_u6_*, and *R_adv_*, have been reported from sunflower and its wild relatives, which are summarized in Ma et al. [5]. Major gene resistance against biotrophic pathogens, such as rust, is generally unstable and nondurable, due to the emergence of virulent races in pathogen populations. In the early 1960s, only four NA *P. helianthi* races, 1, 2, 3, and 4, corresponding to races 100, 500, 300, and 700 of the coded triplet system were identified [6]. Over four decades and more, Gulya and Markell [7] reported 39 NA *P. helianthi* races from 300 rust isolates collected from fields in 2007 and 2008. Friskop et al. [8] identified 29 NA *P. helianthi* races from 238 single-pustule isolates collected from fields in 2011 and 2012. Among the 13 rust *R* genes, only seven, *R_11_*, *R_12_*, *R_13a_*, *R_13b_*, *R_14_*, *R_15_,* and *R_16_*, remain effectively resistant to all *P. helianthi* races identified in the USA [5,9,10,11,12]. In some areas, although a single gene confers resistance to the existing pathogen population, large-scale use of this gene results in the breakdown of resistance. Pyramiding of more than one resistance gene in a single genotype is expected to considerably extend the durability and longevity of resistance due to the low probability of the pathogen being able to assemble multiple, rare virulence genes by mutation or recombination.

In traditional plant breeding, phenotypic selection of superior genotypes within segregating progeny obtained from crosses is a labor-intensive and time-consuming process. Advances in technology have changed agricultural practices over time. Development of modern plant molecular and quantitative genetics over the last three decades has made the integration of biotechnology and conventional breeding possible for many crops. The principles of gene pyramiding assume that parental lines containing target genes and markers linked to the target genes are available. Marker-assisted selection (MAS) can be used to pyramid several *R* genes into a single host genotype, which has been reported in rice [13,14,15], wheat [16,17,18,19,20], barley [21], soybean [22], and tomato [23], as well as in sunflower [24,25].

The rust *R* genes *R_4_* in HA-R3 and *R_5_* in HA-R2 confer resistance to 96.6% and 78.6% of 238 rust isolates tested, respectively, in the USA in 2011 and 2012 [8], while *R_12_* in RHA 464, *R_13b_* in RHA 397, and *R_15_* in HA-R8 confer resistance to all *P. helianthi* races identified in the USA [10]. These genes have been mapped to different sunflower chromosomes corresponding to linkage groups with linked markers—*R_5_* on chromosome 2, *R_15_* on chromosome 8, *R_12_* on chromosome 11, and *R_4_* and *R_13b_* on chromosome 13 [5,10,26,27,28,29]. 

DM is a seedling disease initiated by soil borne oospores of *P. halstedii* or infected seeds. The pathogen infects plants through the roots, eventually becoming systemic. There is no rescue treatment once the disease manifests. The inbred line RHA 464 carries a rust *R* gene, *R_12_*, as well as a broad-spectrum DM *R* gene, *Pl_Arg_*, which is resistant to all *P. halstedii* races [29,30,31,32,33]. *Pl_Arg_* has recently been genetically and physically mapped using high-density single nucleotide polymorphism (SNP) markers on chromosome 1, and the 12 diagnostic SNP markers co-segregating with *Pl_Arg_* span a physical distance of 34.5 Mb, due to suppressed recombination [25]. The distribution of the recombination being population-dependent was reported in wheat [34]. In this study, we report pyramiding of four rust *R* genes, *R_4_*, *R_5_*, *R_13b_*, and *R_15_*, with *R_12_* and *Pl_Arg_* from RHA 464, as well as *R_13b_* and *R_15_*, using MAS to promote rust resistance efficiency and durability. Meanwhile, we examined the possible segregation of cluster markers linked to *Pl_Arg_* in the four distinct F_2_ populations to narrow down the physical interval of *Pl_Arg_*.

## 2. Materials and Methods

### 2.1. Parents and Populations for Gene Pyramiding

Five sunflower lines, HA-R2, HA-R3, HA-R8, RHA 397, and RHA 464, were used in the present study. HA-R2 (PI 650753, carrying the *R_5_* gene) and HA-R3 (PI 650754, carrying *R_4_*) were released in 1985; these are selections from Argentinian open-pollinated cultivars [35]. RHA 397 (PI 597974) and HA-R8 (PI 607511) were released in 1997 and 2001, respectively [36,37]. The *R_13b_* gene in RHA 397 originated from a South African line RO-20-10-3-3-2, while *R_15_* in HA-R8 was derived from a sunflower landrace of PI 432512 collected from Arizona, USA. The RHA 464 (PI 655015) line was released by the USDA-ASR with the North Dakota Agricultural Experiment Station in 2010 and possesses the rust and DM *R* genes *R_12_* and *Pl_Arg_*, respectively [38]. *R_12_* originated from the wild *H. annuus* PI 413047 and *Pl_Arg_* from the wild *H. argophyllus* PI 468651. The sunflower inbred line HA 89, which is susceptible to all DM and rust races was used as a susceptible control in the present study.

Four sunflower lines, HA-R2, HA-R3, RHA 397, and HA-R8, were each crossed with a common parent, RHA 464, to create four F_2_ populations for pyramiding the rust resistance genes combined with DM resistance. Population 1 (Pop1) was derived from a cross between HA-R3 and RHA 464, Population 2 (Pop2) from HA-R2/RHA 464, Population 3 (Pop3) from RHA 397/RHA 464, and Population 4 (Pop4) from HA-R8/RHA 464, while Population 5 (Pop5) was derived from a cross between RHA 397 and HA-R8 for pyramiding of the rust resistance genes *R_13b_* and *R_15_*.

### 2.2. Marker Selection

DNA markers used in the present study are listed in Table 1. The co-dominant nature of the polymorphisms exhibited by the selected markers enables discrimination between homozygotes and heterozygotes in F_2_ populations. In addition, 14 SNP markers diagnostic for *Pl_Arg_* were used for cluster marker analysis. Among them, 12 SNP markers co-segregate with *Pl_Arg_* and physically span a region of 34.5 Mb on chromosome 1 (Table 2) [25]. Marker-assisted selections were performed in all F_2_ generations. For each F_2_ population, initial selection was conducted using one marker per gene, and selected multi-*R* plants were further confirmed with additional markers.

Genomic DNA from each population, along with their parental lines, was extracted from the lyophilized tissues using the DNeasy 96 Plant Kit (Qiagen, Valencia, CA, USA), following the manufacturer’s instructions. DNA quantity and quality were determined using a NanoDrop 2000 Spectrophotometer (Thermo Fisher Scientific, Wilmington, DE, USA). Genotyping of the selected homozygous F_2_ plants from Pop2 and Pop3, along with the SNP markers, were conducted in BioDiagnostics Inc. (River Falls, WI, USA) in 2014. Genotyping of simple sequence repeat (SSR) and PCR-based SNP markers was performed by following the methods described by Qi et al. [9,39]. PCR products were diluted 20–160 times, depending on their yield and were detected using an IR2 4300/4200 DNA Analyzer with denaturing polyacrylamide gel electrophoresis (LI-COR, Lincoln, NE, USA). 

### 2.3. New SSR Marker Development for R_15_

The rust resistance gene *R_15_* was recently mapped to the upper end of sunflower chromosome 8 [5]. Unfortunately, SNP markers linked to *R_15_* did not have any polymorphisms between HA-R8 and RHA 464. We extracted a sequence from the sunflower reference genome HA412-HO using the flanking markers SFW05824 (physical position 10,040,792 bp) and NSA_008457 (11,391,650 bp). SSRs were identified from the extracted sequences using the SSR Identification Tool from the Gramene. Out of the 13 designed SSR markers, two SSRs, SUN398 and SUN406, that mapped close to *R_15_* and were used to screen *R_15_* in Pop4 derived from the HA-R8/RHA 464 cross and the Pop5 derived from HA-R8/RHA 397. The SSR primer sequences were as follows (5′–3′): SUN398 F-ATCCAACCCGACTTCTTCGG, R-TGACAAACAGCCGCCTCTC and SUN406 F-CTCACTGGAAGCAGCCTCTC, R-TTCCATGTGCATCAATGTGGC.

### 2.4. Disease Evaluation

In addition to DNA marker selection in the F_2_ generations, both DM and the rust-resistant tests of these F_2_-derived F_3_ families that had two or three genes of interest in the homozygous state were conducted under greenhouse control conditions. The whole seedling immersion method was applied to test reactions to DM in sunflower seedlings using the NA *P. halstedii* race 734 [39,40], a new virulent race identified in the USA in 2010 [41]. Sunflower seedlings infected with DM display typical leaf chlorosis with white sporulation on the underside of their cotyledons and true leaves. A plant was scored as susceptible (S) if sporulation was observed on the cotyledons and true leaves, and was scored as resistant (R) if no sporulation was observed.

Twenty-four to seventy-two individual seedlings of each F_3_ family were first inoculated with *P. halstedii* race 734, and the resistant plants were then transferred to 36 cell plastic flats (each cell 4.6 cm × 5.4 cm) filled with Sunshine SB 100B potting mixture (SunGro Horticulture, Bellevue, WA, USA). After 10–12 days, seedlings at the four-leaf stage were inoculated with *P. helianthi* race 336, as described by Qi et al. [9]. The infection types (ITs) of rust were recorded on a scale of 0–4 [42], combined with the percentage of leaf area covered in pustules (severity), as described by Gulya et al. [43], after 12–14 days post inoculation. IT 0, 1, and 2 along with pustule coverage of 0 to 0.5% were recorded as resistant, while IT 3 and 4 with pustule coverage larger than 0.5% were considered susceptible.

## 3. Results

### 3.1. Marker Selection and Disease Evaluation of Homozygous Multi-Resistant Plants

#### 3.1.1. *R_4_*/*R_12_*/*Pl_Arg_* Homozygous Plants

**Figure 1:**
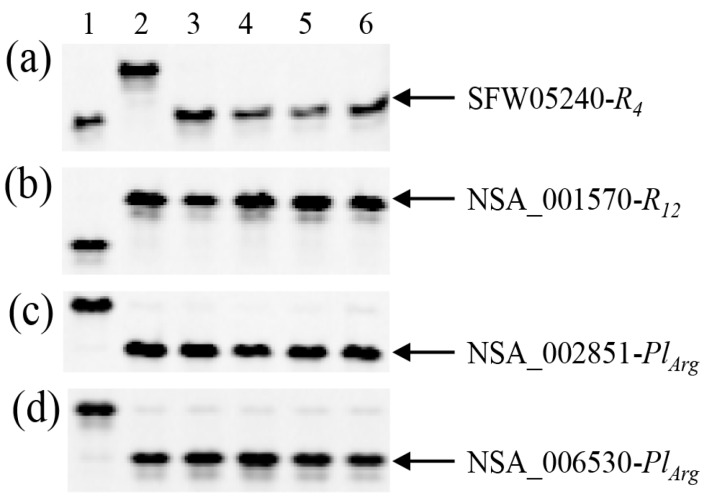
PCR gel image of single nucleotide polymorphism (SNP) markers for testing homozygous triple-resistant F_2_ plants from HA-R3/RHA 464. (**a**) SFW05240 linked to *R_4_*, (**b**) NSA_001570 linked to *R_12_*, (**c**,**d**) NSA_002851 and NSA_006530 linked to *Pl_Arg_*. 1: HA-R3, 2: RHA 464, 3–6: Homozygous triple-resistant plants for *R_4_*/*R_12_*/*Pl_Arg_*.

Three DNA markers, ORS316, NSA_001392, and NSA_002798, linked to genes *R_4_*, *R_12_*, and *Pl_Arg_*, respectively, were first used to screen Pop1, which was derived from a cross between HA-R3 and RHA 464, for genotypes being homozygous for the three genes. Out of the 376 F_2_ individuals screened, four plants were selected as homozygotes at all three marker loci and were further confirmed by the additional six markers, SFW05240 and SFW01497 for *R_4_*, NSA_00064 and NSA_001570 for *R_12_*, and NSA_002851 and NSA_006530 for *Pl_Arg_* (Figure 1, Table 1).

To confirm the presence of DM and rust resistance and to observe the effects of stacking rust genes, DM and rust tests were performed on selected F_2_-derived F_3_ families. The susceptible line HA 89 and the parental line HA-R3 showed the expected susceptibility reactions to NA *P. halstedii* race 734 exposure, after seedling inoculation, while 197 F_3_ plants from four F_2_-derived F_3_ families exhibited resistance to race 734 (Table 3). Subsequent rust tests revealed that F_3_ plants with two gene stacks, *R_4_* and *R_12_*, were highly resistant, showing hypersensitive fleck without pustules after seedling inoculation with *P. helianthi* race 336, as compared to the parental lines HA-R3 and RHA 464, which exhibited an IT of 1 and a pustule coverage of 0.1% (Table 3). These results indicated an enhanced rust resistance in these plants. DM and rust tests confirmed that the entries were homozygous for *R_4_*, *R_12_*, and *Pl_Arg_*.

#### 3.1.2. *R_5_*/*R_12_*/*Pl_Arg_* Homozygous Plants

A total of 752 F_2_ individuals from Pop2, derived from the HA-R2/RHA 464 cross, were initially screened using three markers. The SSR marker ORS1197 was used to identify F_2_ plants with the *R_5_* gene, SNP NSA_001392 for *R_12_*, and SSR ORS610 for *Pl_Arg_*. Four plants with homozygous alleles at all three gene loci (*R_5_ R_5_*/*R_12_R_12_*/*Pl_Arg_Pl_Arg_*) were identified and further confirmed with seven additional SNP markers, three (SFW03654, NSA_000267, and NSA_001605) linked to *R_5_*, two (NSA_000064 and NSA_001570) linked to *R_12_*, and two (NSA_002851 and NSA_006530) linked to *Pl_Arg_* (Table 1).

DM phenotyping indicated that, as expected, the parental line HA-R2 was susceptible to *P. halstedii* race 734, similar to HA 89, a susceptible control, while 232 plants from the four F_3_ families exhibited homozygous resistance to downy mildew like the resistant donor RHA 464 (Table 3), confirming that these entries were homozygous for *Pl_Arg_*. In subsequent rust tests, the susceptible control HA 89 line developed severe symptoms with an IT of 4 and a pustule coverage of 40%, after infection with *P. helianthi* race 336 (Table 3). Comparing the effect of the different resistance sources for rust disease, the effect of the rust resistance gene in RHA 464 was higher than that in HA-R2. HA-R2 had an IT of 2 with a pustule coverage of 0.5%, while RHA 464 had an IT of 1 with a pustule coverage of 0.1% (Table 3). The selected plants with two gene stacks, *R_5_* and *R_12_*, had a reaction to rust infection similar to RHA 464.

#### 3.1.3. *R_13b_*/*R_12_*/*Pl_Arg_* Homozygous Plants

Initial screens of Pop3, derived from the RHA 397/RHA 464 cross, were conducted using three markers, ORS316 for *R_13b_*, NSA_001392 for *R_12_*, and ORS610 for *Pl_Arg_*, and three of the 758 F_2_ plants tested were homozygous at all three marker loci. The selected triple *R*-plants were further confirmed with six additional SNP markers, three NSA_000187, NSA_005565, and NSA_006846, one NSA_001570, and two NSA_002851 and NSA_006530 linked to the three targeted genes, respectively (Table 1). 

As expected, the susceptible control HA 89 and the parental RHA 397 lines were susceptible to downy mildew, while the 180 F_3_ individuals from the three F_2_-derived F_3_ families, along with their resistant donor RHA 464, were resistant to inoculation with *P. halstedii* race 734. All F_3_ plants carrying *R_13b_* and *R_12_* proved to be free of rust infection after inoculation with *P. helianthi* race 336 (Table 3). This demonstrated that the combination of *R_13b_* and *R_12_* exerted an additive effect on the degree of resistance to rust.

#### 3.1.4. *R_15_*/*R_12_* Homozygous Plants 

Two new SSR markers were developed in the present study due to a lack of polymorphic SNP markers linked to *R_15_* in the HA-R8/RHA 464 F_2_ population. Linkage analysis with 186 F_2_ segregating plants derived from the HA 89/HA-R8 cross previously used to map the *R_15_* gene [5] indicated that SSRs SUN398 and SUN406 co-segregated with a previously mapped SNP marker, SFW05824, distal to *R_15_* at a genetic distance of 0.4 cM (Table 1).

A total of 470 F_2_ plants from HA-R8/RHA 464 were first screened using three markers, SSR SUN398, SNPs NSA_001392, and NSA_002798, targeting three genes, *R_15_*, *R_12_*, and *Pl_Arg_*, respectively, and two plants, 16-46-202 and 16-46-329, were identified as homozygous for all three loci. The three additional markers, SUN406, NSA_001570, and NSA_001835, one for each gene, further confirmed their homozygous state (Table 1). 

Unexpectedly, 92 F_3_ plants from the two F_2_-derived F_3_ families exhibited homozygous susceptibility to DM, similar to the susceptible parent HA-R8, after inoculation with *P. halstedii* race 734 (Table 3). *Pl_Arg_* in RHA 464 co-segregated with 12 SNP markers that spanned a region of 34.5 Mb on chromosome 1 (Table 2) [25]. The two SNP markers used in the above screening, NSA_002798 and NSA_001835, were located on the lower end of the marker cluster (Table 2). One possibility is that recombination occurred between the clustered markers and the gene during line development, which altered the linkage phase between the markers and *Pl_Arg_*. To confirm this hypothesis, we tested these 14 SNP markers in all selected F_2_ individuals from the four F_2_ populations, from which the F_3_ families were derived (see below).

Because the F_3_ families tested were susceptible to DM and died, we regrew 44 and 48 pyramiding individuals from each of the two F_3_ families carrying *R_12_* and *R_15_* for rust evaluation. No segregation was detected in the rust phenotypic assessment, indicating the homozygous state of the selected F_3_ families. All F_3_ plants exhibited hypersensitive fleck without pustules after seedling inoculation with *P. helianthi* race 336, indicating an increased resistance to rust, as compared to both parents (Table 3). 

#### 3.1.5. *R_13b_*/*R_15_* Homozygous Plants 

**Figure 2:**
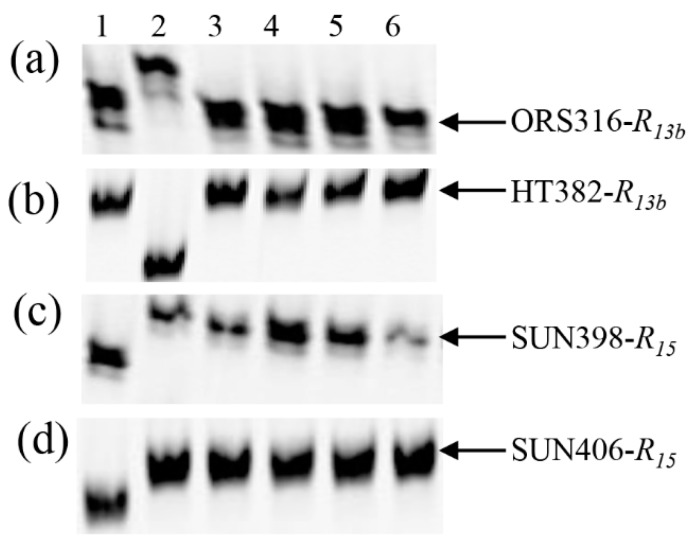
PCR gel image of SSR markers for testing the homozygous double-resistant F_2_ plants from RHA397/HA-R8. (**a**,**b**) ORS316 and HT382 linked to *R_13b_*. (**c**,**d**) SUN398 and SUN406 linked to *R_15_*. 1: RHA 397, 2: HA-R8, 3–6: Homozygous double-resistant plants for *R_13b_*/*R_15_*.

Two SSR markers, ORS316 and SUN398, targeting the rust *R* genes *R_13b_* and *R_15_*, respectively, were used to screen Pop5, derived from the RHA 397 and HA-R8 cross. Twenty plants from 376 F_2_ individuals tested were homozygous at both marker loci, which was confirmed by an additional two markers, HT382 for *R_13b_* and SUN406 for *R_15_* (Figure 2). 

A total of 192 F_3_ plants from the four selected F_3_ families were evaluated for rust resistance using the *P. helianthi* race 336, along with the susceptible control HA 89 and both parents, RHA 397 and HA-R8. All F_3_ plants exhibited a hypersensitive fleck without pustules, compared to the susceptible control HA 89, which had an IT of 4, and more than 40% of the leaves were covered with pustules, and both parents, which had an IT of 1% and 0.1% of leaves, were covered with pustules (Table 3).

### 3.2. Detection of Recombination in the Marker Cluster Linked to Pl_Arg_

**Figure 3:**
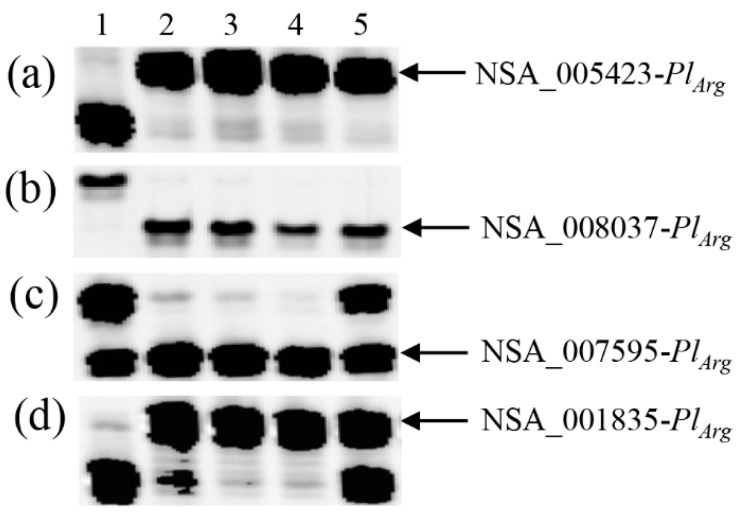
PCR gel image of SNP markers linked to *Pl_Arg_* indicates recombination between NSA_008037 and NSA_007595 in selected F_2_ plants from RHA 397/RHA 464. (**a**) NSA_005423, (**b**) NSA_008037, (**c**) NSA_007595, and (**d**) NSA_001835. 1: RHA 397, 2: RHA 464, 3: 14-21-319, 4: 14-21-413, 5: 14-21-129. 14-21-129 was homozygous at the NSA_005423 and NSA_008037 loci but heterozygous at the NSA_007595 and NSA_001835 loci.

A total of 14 SNP markers that are diagnostic for RHA 464 marker alleles linked to *Pl_Arg_* were used to detect recombination among cluster markers in the multi-resistant F_2_ plants selected from the four different F_2_ populations. Of the 14 SNP markers selected, 12, spanning a region of 34.5 Mb on chromosome 1 physical map, co-segregated with *Pl_Arg_*, and two were proximal to *Pl_Arg_*, with genetic distances of 0.31 and 0.83 cM (Table 2) [25]. No recombination was detected in F_2_ plants derived from Pop1 of the HA-R3/RHA 464 cross or Pop2 of HA-R2/RHA 464 (Table 4). In Pop3 of RHA 397/RHA 464, one (14-21-129) of the three F_2_ plants exhibited recombination between markers NSA_008037 and NSA_007595, based on their physical positions (Figure 3, Table 4). The 14-21-129 F_2_ plant had heterozygous alleles at three SNP loci, NSA_007595, NSA_001835, and NSA_006530, while the F_2_-derived F_3_ family exhibited homozygous resistance to DM, indicating that the recombination did not involve the *Pl_Arg_* locus (Table 3).

**Figure 4:**
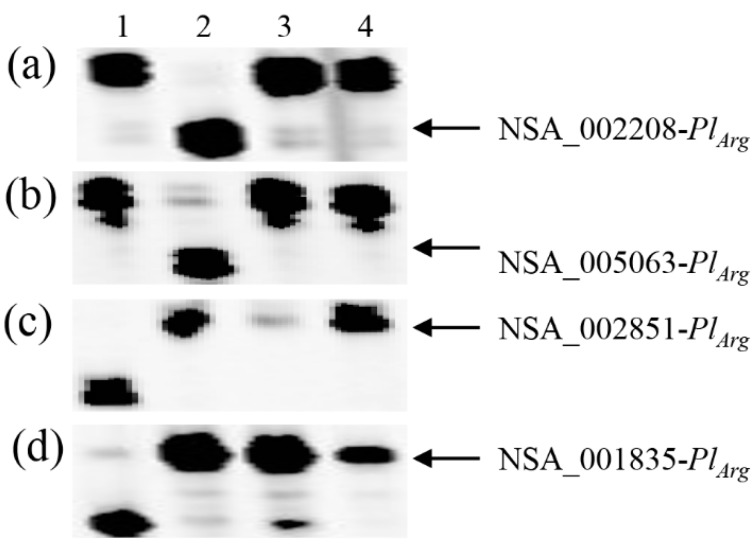
PCR gel image of SNP markers linked to *Pl_Arg_* indicates recombination between NSA_005063 and NSA_002851 in the selected F_2_ plants from HA-R8/RHA 464. (**a**) NSA_002208, (**b**) NSA_005063, (**c**) NSA_002851, and (**d**) NSA_001835. 1: HA-R8, 2: RHA 464, 3: 16-46-202, and 4: 16-46-329. 16-46-202 and 16-46-329 did not have *Pl_Arg_* SNP alleles at the NSA_002208 or NSA_005063 loci but had *Pl_Arg_* SNP alleles at the NSA_002851 and NSA_001835 loci.

**Figure 5:**
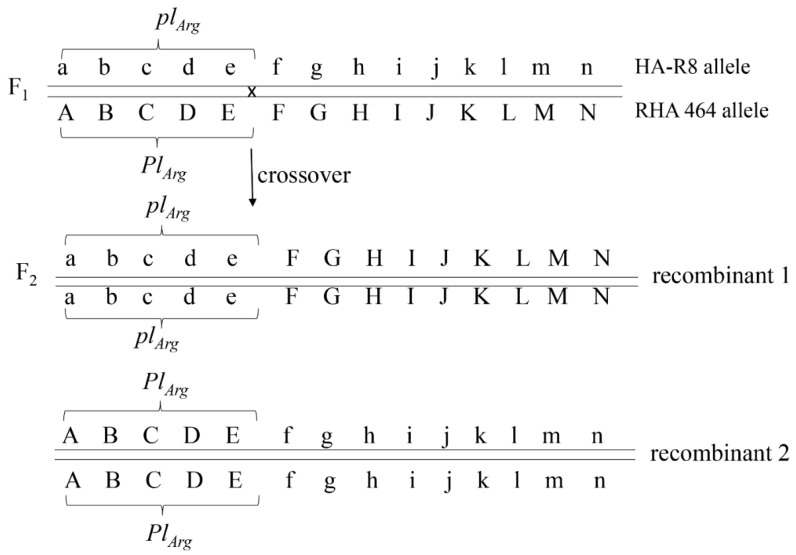
A crossover occurred between marker E and F and changed the linkage phase of *Pl_Arg_* with the markers. A to N represent 14 SNP markers listed in Table 2. Lower case letters represent the HA-R8 SNP allele and the upper case letters represent the RHA 464 SNP allele. *Pl_Arg_* co-segregated with the first five markers.

In Pop4, derived from the HA-R8/RHA 464 cross, recombination was detected between SNP markers NSA_005063 and NSA_002851 based on their physical position in the two selected F_2_ plants, 16-46-202 and 16-46-329 (Figure 4, Table 2 and Table 4). Five SNPs, NSA_002208, NSA_000630, NSA_004149, NSA_005423, and NSA_005063, physically located in a region between 106 and 123 Mb, were homozygous for the HA-R8 SNP alleles, while the remaining nine SNPs physically located in a region between 124 and 145 Mb were homozygous for the RHA 464 SNP alleles. Two SNP markers, NSA_002798 and NSA_001835, which were used for the initial selection of F_2_ plants, belonged to the latter group. Although the two selected F_2_ plants had RHA 464 alleles at both NSA_002798 and NSA_001835 loci, the F_2_-derived F_3_ families exhibited homozygous susceptibility to DM, indicating that the genetic linkage between the markers and the *Pl_Arg_* gene was broken, altering the linkage phase of *Pl_Arg_* with the markers. Combined phenotyping and genotyping data placed *Pl_Arg_* in a position close to the first five SNP markers in the cluster (Figure 5, Table 4). 

## 4. Discussion

Marker-assisted gene pyramiding has previously been successfully used in plant breeding, especially when selecting for disease and insect resistance controlled by major genes; for review see [44]. In the present study, we developed five pyramids with different rust *R* gene combinations, three of which were combined with a DM *R* gene: *Pl_Arg_*, *R_4_*/*R_12_*/*Pl_Arg_*, *R_5_*/*R_12_*/*Pl_Arg_*, *R_13b_*/*R_12_*/*Pl_Arg_*, *R_15_*/*R_12_*, and *R_13b_*/*R_15_*. Accumulating major genes for resistance in an elite genotype by conventional breeding is laborious and time-consuming when one or more of the genes are effective against all known isolates of the pathogen. Due to a lack of *P. helianthi* race to differentiate the *R_12_* gene from the other four rust *R* genes, selection for plants having multiple genes using molecular markers is extremely important. The co-dominant nature of both SSR and SNP markers used in this study made it possible to select homozygous pyramids in the F_2_ generation. Rust evaluation of the F_2_-derived F_3_ families indicated that the pyramids generally showed enhanced resistance to the *P. helianthi* pathogen, compared to the parental lines, demonstrating a complementary effect of the two *R* genes when present together. The pyramids carrying *R_4_*/*R_12_*/*Pl_Arg_*, *R_13b_*/*R_12_*/*Pl_Arg_*, *R_15_*/*R_12_*, and *R_13b_*/*R_15_* proved to be free of rust infection (Table 3). These lines, once released, will serve as valuable germplasms for the breeder to use in breeding programs. As a hybrid crop, sunflower breeders can transfer the different gene combinations into the cytoplasm male sterile (CMS) and male fertility restorer (Rf) lines, respectively, and the resulting F_1_ hybrids from the crosses of CMS/Rf will carry multiple rust *R* genes and a DM *R* gene, effectively suppressing the emergence of virulent isolates of the rust pathogen and potentially providing wider spectra and durable resistance to rust.

The minimum population size for successful recovery of a desirable genotype can be calculated in a three-unlinked gene pyramiding project. To obtain one F_2_ individual that is homozygous for resistance alleles at all three gene loci with a 99% probability of success, 293 individuals must be evaluated [45]. In the present study, we screened the four F_2_ populations, with sizes ranging from 376 to 758 F_2_ individuals, recovering three-gene pyramids from Pop1 (4/376), Pop2 (4/756), and Pop3 (3/758); Pop4 was an exception. As the recombination occurred between the flanking markers of *Pl_Arg_*, two selected F_2_ plants from 470 F_2_ individuals had lost the *Pl_Arg_* gene (Table 4).

*Pl_Arg_* was originally transferred from a wild *H. argophyllus* into cultivated sunflower in 1989 with no reports of resistance breakdown for more than 25 years [31,33,46]. Molecular mapping placed *Pl_Arg_* in a region with highly suppressed recombination on sunflower chromosome 1 [30,47]. Qi et al. [25] reported that 78 SNP markers co-segregated with *Pl_Arg_* in an F_2_ population derived from the cross of HA 89/RHA 464, and 12 of them were diagnostic for *Pl_Arg_*, which spanned a physical distance of 34.5 Mb (between 106.0 Mb and 140.5 Mb) in the HA412-HO genome assembly (Table 2). In the present study, the recombination events were observed in the marker cluster in the two F_2_ populations of RHA 397/RHA 464 and HA-R8/RHA 464. Crossover occurred between NSA_008037 and NSA_007595 in plant 14-21-129 in Pop3 of RHA 397/RHA 464 but did not change the linkage phase of *Pl_Arg_* with the markers (Table 4). Plant 14-21-129 displayed heterozygous alleles in three marker loci, NSA_007595, NSA_001835, and NSA_006530, while the F_3_ family derived from 14-21-129 exhibited homozygous resistance to DM. However, the observed recombination between NSA_005063 and NSA_002851 in Pop4 of HA-R8/RHA 464 did change the linkage phase of the *Pl_Arg_* with markers. Of the 14 SNP markers tested, selected F_2_ plants showed HA-R8 SNP alleles in the first five SNP loci and RHA 464 SNP alleles in the latter nine SNP loci (Table 2 and Table 4). The F_2_-derived F_3_ families were all susceptible to DM, indicating that *Pl_Arg_* is close to the first five SNPs in the marker cluster (Figure 5). This finding narrows down the *Pl_Arg_*-harboring region from 34.5 Mb to 17.3 Mb—between 106.0 Mb and 123.3 Mb (Table 2). 

A high-density SNP map for *Pl_Arg_* was constructed using a biparental F_2_ population, as is the case for most genetic maps generated where one cycle of meiosis provided all recombination events in the population [25]. Whereas, a breeding program might involve more than two parents and multiple crosses, as a result, greatly increasing the recombination rate in breeding population and decreasing the reliability of the marker-based selection because of the increasing cycles of meiosis. Although the present study placed *Pl_Arg_* within a five-SNP cluster that co-segregated with the gene, these markers still spanned a physical distance of 17.3 Mb. With potential increases in recombination in breeding populations, there is a chance that crossover occurred between *Pl_Arg_* and the five SNP-cluster. Using SNP markers selected from a five SNP-cluster combining the closest flanking marker, NSA_002851, is recommended in MAS for breeding programs, which would greatly increase the reliability of the markers for predicting phenotypes.

## Figures and Tables

**Table 1:** DNA markers used in the present study.

Markers/Genes	Marker Type	Chromosome/Linkage Group	Position (cM)	Reference
**ORS316**	SSR	13	3.5	[28]
SFW05240	SNP		3.5	
*R_4_*			4.1	
SFW01497	SNP		4.8	
**ORS1197**	SSR	2	12.2	[28]
NSA_001605	SNP		14.4	
SFW03654	SNP		14.9	
*R_5_*			15.5	
NSA_000267	SNP		16.7	
NSA_000064	SNP	11	44.6	[32]
*R_12_*			45.4	
**NSA_001392**	SNP		46.8	
NSA_001570	SNP		46.8	
**ORS316**	SSR	13	5.9	[28]
NSA_000187	SNP		5.9	
NSA_005565	SNP		5.9	
NSA_006846	SNP		5.9	
*R_13b_*			6.8	
HT382	SSR		14.4	
**SUN398**	SSR	8	17.7	[5], present study
SUN406	SSR		17.7	
*R_15_*			18.1	
**ORS610**	SSR	1	29.4	[25]
*Pl_Arg_*			29.7	
NSA_002851	SNP		29.7	
**NSA_002798**	SNP		29.7	
NSA_001835	SNP		30.0	
NSA_006530	SNP		30.5	

Markers used for initial screening are in bold.

**Table 2:** SNP markers and their genetic and physical position in relation to *Pl_Arg_*.

SNP ID	Recombination between Markers ^a^	Genetic Position (cM) ^a^	Physical Position (bp) in HA 412-HO Assembly	
			Start	End
NSA_002208		29.68	105,999,004	105,999,300
NSA_000630	0	29.68	108,193,829	108,194,264
NSA_004149	0	29.68	109,941,814	109,942,141
NSA_005423	0	29.68	110,935,770	110,936,060
NSA_005063	0	29.68	123,281,156	123,281,926
***Pl_Arg_***	0	29.69		
NSA_002851	0	29.68	124,006,083	124,006,420
NSA_002867	0	29.68	129,149,732	129,150,065
NSA_005624	0	29.68	132,990,525	132,990,886
NSA_002798	0	29.68	135,732,577	135,733,004
NSA_002131	0	29.68	135,852,458	135,852,909
NSA_008037	0	29.68	137,830,356	137,830,737
NSA_007595	0	29.68	140,507,251	140,507,591
NSA_001835	1	30.00	143,343,690	143,344,747
NSA_006530	1	30.52	143,859,036	143,859,455

^a^ Modified from Qi et al. (2017) based on the current study. The size of the sunflower chromosome 1 physical map is 175,985,764 bp for HA412-HO assembly.

**Table 3:** Summary of Downy Mildew and Rust Tests in F_3_ Families.

Plant No.	Genes/Pyramided Genes	Materials	DM Score (Race 734)			Rust Score (Race 336)		
			No. of Plants Tested	S	R	No. of Plants Tested	IT	Severity
2008 GH	-	HA 89 (S-control)	12	12	0	12	4	40
2014 GH	*R_4_*	HA-R3	24	24	0	12	1	0.1
15-2076	*R_12_*/*Pl_Arg_*	RHA 464	12	0	12	12	1	0.1
16-069-18	*R_4_*/*R_12_*/*Pl_Arg_*	HA-R3 × RHA 464 F_3_	36	0	36	36	0	0
16-069-46			60	0	60	60	0	0
16-069-121			53	0	53	53	0	0
16-069-288			60	0	60	60	0	0
2008 GH	-	HA 89 (S-control)	12	12	0	12	4	40
2012 GH	*R_5_*	HA-R2	20	20	0	12	2	0.5
15-2076	*R_12_*/*Pl_Arg_*	RHA464	12	0	12	12	1	0.1
14-22-693	*R_5_*/*R_12_*/*Pl_Arg_*	HA-R2 × RHA 464 F_3_	54	0	54	54	1	0.1
14-22-694			46	0	46	46	1	0.1
14-22-737			68	0	68	68	1	0.1
14-22-786			64	0	64	64	1	0.1
2008 GH	-	HA 89 (S-control)	12	12	0	12	4	40
10-002-2	*R_13b_*	RHA 397	16	16	0	12	1	0.1
15-2076	*R_12_*/*Pl_Arg_*	RHA 464	12	0	12	12	1	0.1
14-21-129	*R_13b_*/*R_12_*/*Pl_Arg_*	RHA 397 × RHA 46 F_3_	48	0	48	48	0	0
14-21-319			72	0	72	72	0	0
14-21-413			60	0	60	60	0	0
2008 GH	-	HA 89 (S-control)	12	12	0	12	4	40
2012 GH	*R_15_*	HA-R8	28	28	0	12	1	0.1
15-2076	*R_12_*/*Pl_Arg_*	RHA 464	12	0	12	12	1	0.1
16-46-202	*R_15_*/*R_12_*	RHA 464 × HA-R8 F_3_	32	32	0	44	0	0
16-46-329			28	28	0	48	0	0
2008 GH	-	HA 89 (S-control)	-	-	-	32	4	40
2012 GH	*R_15_*	HA-R8	-	-	-	16	1	0.1
10-002-2	*R_13b_*	RHA 397	-	-	-	16	1	0.1
16-043-13	*R_13b_*/*R_15_*	RHA 397 × HA-R8 F_3_	-	-	-	48	0	0
16-043-115			-	-	-	48	0	0
16-044-81			-	-	-	48	0	0
16-044-181			-	-	-	48	0	0

S, susceptible; R, resistant.

**Table 4:** Summary of the *Pl_Arg_* cluster marker tests in the multi-resistant F_2_ plants selected from the four F_2_ populations.

Selected F_2_ Plants	F_3_ DM Phenotype	*Pl_Arg_* SNP Marker													
		NSA_002208	NSA_000630	NSA_004149	NSA_005423	NSA_005063	NSA_002851	NSA_002867	NSA_005624	NSA_002798	NSA_002131	NSA_008037	NSA_007595	NSA_001835	NSA_006530
RHA397	S	A	A	A	A	A	A	A	A	A	A	A	A	A	A
RHA464	R	B	B	B	B	B	B	B	B	B	B	B	B	B	B
14-21-129	R	B	B	B	B	B	B	B	B	B	B	**B**	**H**	H	H
14-21-319	R	B	B	B	B	B	B	B	B	B	B	B	B	B	B
14-21-413	R	B	B	B	B	B	B	B	B	B	B	B	B	B	B
HA-R2	S	A	A	A	A	A	A	A	A	A	A	A	A	A	A
RHA464	R	B	B	B	B	B	B	B	B	B	B	B	B	B	B
14-22-693	R	B	B	B	B	B	B	B	B	B	B	B	B	B	B
14-22-694	R	B	B	B	B	B	B	B	B	B	B	B	B	B	B
14-22-737	R	B	B	B	B	B	B	B	B	B	B	B	B	B	B
14-22-786	R	B	B	B	B	B	B	B	B	B	B	B	B	B	B
HA-R3	S	A	A	A	A	A	A	A	A	A	A	A	A	A	A
RHA464	R	B	B	B	B	B	B	B	B	B	B	B	B	B	B
16-69-18	R	B	B	B	B	B	B	B	B	B	B	B	B	B	B
16-69-46	R	B	B	B	B	B	B	B	B	B	B	B	B	B	B
16-69-121	R	B	B	B	B	B	B	B	B	B	B	B	B	B	B
16-69-288	R	B	B	B	B	B	B	B	B	B	B	B	B	B	B
HA-R8	S	A	A	A	A	A	A	A	A	A	A	A	A	A	A
RHA464	R	B	B	B	B	B	B	B	B	B	B	B	B	B	B
16-46-202	S	A	A	A	A	**A**	**B**	B	B	B	B	B	B	B	B
16-46-329	S	A	A	A	A	**A**	**B**	B	B	B	B	B	B	B	B

A, SNP allele other than that of RHA 464; B, RHA 464 SNP allele; H, heterozygous; S, homozygous susceptible; R, homozygous resistant. The bold capital letters indicate recombination between markers.
